# Cerebral white matter burden is linked to cognitive function in patients undergoing hemodialysis

**DOI:** 10.1080/07853890.2024.2310142

**Published:** 2024-02-07

**Authors:** Tsai-Shan Wu, Ping-Hsun Wu, Hsiu-Fen Lin, Wen-Ching Chen, Teng-Hui Huang, Ming-Yen Lin, Yun-Shiuan Chuang, Fan-Pei Gloria Yang, Yi-Wen Chiu, Jer-Ming Chang, Mei-Chuan Kuo, Yi-Ting Lin

**Affiliations:** a Department of General Medicine, Kaohsiung Medical University Hospital, Kaohsiung Medical University, Kaohsiung, Taiwan; b Division of Nephrology, Department of Internal Medicine, Kaohsiung Medical University Hospital, Kaohsiung, Taiwan; c Faculty of Medicine, College of Medicine, Kaohsiung Medical University, Kaohsiung, Taiwan; d Center for Big Data Research, Kaohsiung Medical University, Kaohsiung City, Taiwan; e Department of Neurology, Kaohsiung Medical University Hospital, Kaohsiung Medical University, Kaohsiung, Taiwan; f Department of Family Medicine, Kaohsiung Medical University Hospital, Kaohsiung, Taiwan; g Department of Foreign Languages and Literature, National Tsing Hua University, Hsinchu, Taiwan; h Center for Cognition and Mind Sciences, National Tsing Hua University, Hsinchu, Taiwan; i Department of Radiology, Graduate School of Dentistry, Osaka University, Suita, Japan

**Keywords:** Brain magnetic resonance imaging, cognitive function, Fazekas scale, hemodialysis

## Abstract

**Introduction:**

Chronic kidney disease is related to neurodegeneration and structural changes in the brain which might lead to cognitive decline. The Fazekas scale used for assessing white matter hyperintensities (WMHs) was associated with poor cognitive performance. Therefore, this study investigated the associations between the mini-mental status examination (MMSE), Montreal cognitive assessment (MoCA), cognitive abilities screening instrument (CASI), and Fazekas scale in patients under hemodialysis (HD).

**Methods:**

The periventricular (PV) WMHs and deep WMHs (DWMHs) in brain magnetic resonance images of 59 patients under dialysis were graded using the Fazekas scale. Three cognition function tests were also performed, then multivariable ordinal regression and logistic regression were used to identify the associations between cognitive performance and the Fazekas scale.

**Results:**

There were inverse associations between the three cognitive function tests across the Fazekas scale of PVWMHs (*p* = .037, .006, and .008 for MMSE, MoCA, and CASI, respectively), but the associations were attenuated in the DWMHs group. In CASI, significant differences were identified in short-term memory, mental manipulation, abstract thinking, language, spatial construction, and name fluency in the PVWMHs group. However, DWMHs were only significantly correlated with abstract thinking and short-term memory.

**Conclusion:**

An inverse correlation existed between the Fazekas scale, predominantly in PVWMHs, and cognition in patients undergoing HD. The PVWMHs were associated with cognitive performance assessed by MMSE, MoCA, and CASI, as well as with subdomains of CASI such as memory, language and name fluency in patients undergoing HD.

## KEY MESSAGES

An inverse correlation existed between the Fazekas scale and cognition in patients undergoing hemodialysis, predominantly in periventricular white matter hyperintensities.

The periventricular white matter hyperintensities were associated with cognitive performance assessed by mini-mental status examination (MMSE), Montreal cognitive assessment (MoCA), cognitive abilities screening instrument (CASI), as well as with subdomains of CASI such as memory, language and name fluency in patients undergoing HD.

## Introduction

Chronic kidney disease (CKD) is a growing public health concern worldwide and is associated with increased risk for multiple adverse outcomes, including cognitive impairments, cardiovascular diseases, and death [1]. Patients with CKD have a reported prevalence of cognitive impairment ranging from 13 to 70% [2]. Various assessment tools such as the Mini-Mental State Exam (MMSE), the Trails Making Tests (TMT) forms A and B and components of the Wechsler Adult Intelligence Scale (WAIS), the 6-item cognitive impairment test, digit symbol substitution test, Montreal Cognitive Assessment (MoCA) and Cognitive Abilities Screening Instrument (CASI) have been used for detecting or quantifying the magnitude of the dysfunction [3,4]. Certain screening neuropsychological assessments with specific subdomains such as MMSE, TMT forms A, and MoCA can characterize specific cognitive impairments with better validity; though MMSE is the most commonly used and MoCA is the preferred tool for patients with kidney disease due to better prediction of cognitive impairment in patients with CKD [2–4]. Previous studies focusing on non-dialytic CKD patients suggested the dysfunctions involved several domains such as orientation and attention, naming, processing speed, memory, language, and executive functions [5–7]. A systematic review and meta-analysis [8] reported that end-stage kidney disease (ESKD) patients undergoing hemodialysis (HD) had impaired cognitive functions, particularly in the domains of orientation and attention and executive functions compared to the general population.

White matter hyperintensities (WMHs), a common finding in Magnetic Resonance Imaging (MRI) in the elderly, are linked to ischemic alterations from small vessel disease. These hyperintensities are commonly attributed to conditions such as vascular dementia and stroke [9]. The Fazekas scale, known for ranking WMHs [10], is used to assess the amount of white matter changes in brain MRI scans. The scale categorizes periventricular (PV) WMHs and deep WMHs (DWMHs) into four grades, and it has been linked to cognitive function decline [11–17]. Among studies supporting the correlation, Sudo et al. [11] reported the correlation between the modified Fazekas scale and performances in TMT B (time to complete and errors), difference TMT B-A and CAMCOG (Cambridge Cognition Examination) total score in vascular mild cognitive impairment patients. Moreover, another study also supported that extensive WMHs corresponded with cognitive impairment in attention, memory, social cognition, and subjective cognitive performance when the Fazekas scale and DSM-5 were used [15].

Although there may be a potential link between WMHs and reduced cognitive function in certain domains, the relationship between WMHs and cognitive function assessment in patients with kidney disease has not been comprehensively assessed. Therefore, the associations between different Fazekas stages of WMHs and cognitive function by applying the Mini-Mental State Examination (MMSE), Montreal Cognitive Assessment (MoCA), and Cognitive Abilities Screening Instrument (CASI) were investigated in patients undergoing HD.

## Materials and methods

### Hemodialysis participants

The patients undergoing HD were recruited from August 2016 to January 2017 in two HD units at Kaohsiung Medical University Hospital and Kaohsiung Municipal Hsiao-Kang Hospital in Kaohsiung, Taiwan. Eligible criteria were patients who were at least 30 years old and had been on maintenance dialysis for at least 90 days. All patients underwent regular HD three times per week using automated volumetric equipment and high-flux dialyzers which lasted between 3.5 and 4 h. The dialysate flow was kept at 500 ml/min and the blood flow was maintained between 250 and 300 ml/min, with the single pool Kt/V greater than 1.2 per week. The exclusion criteria encompassed patients who had experienced an acute stroke within the past three months, those with space-occupying lesions in the brain, individuals with a history of depression, psychosis, or substance use disorder, those with a seizure disorder as well as anyone with contraindications to undergoing an MRI. The Kaohsiung Medical University Institutional Review Board (KMUHIRB-E(I)-20160095) approved the study protocol and all participants provided written informed consent. Cognitive function was assessed using the MMSE, MoCA, and CASI.

### Comorbidity, laboratory, and clinical variables

Sociodemographic data (age, gender, and years of education), time on dialysis, medical history, and biochemical data were obtained through electronic healthcare system records. Diabetes mellitus was defined as an HbA1C ≥6.5% or taking diabetes medications and cerebrovascular disease was defined as a history of a cerebrovascular incident due to hemorrhage or infarction.

The patients fasted overnight before blood sampling and samples were collected *via* the arteriovenous fistula or graft immediately before the patients’ scheduled HD session at a single midweek dialysis session. Serum hemoglobin and albumin were available for patients undergoing HD from regular blood samples acquired at the start of the HD session within 30 d before cognitive testing.

### Brain image acquisition and Fazekas scale evaluation

MRI scanning of the brain was performed with a 3.0-Tesla MRI scanner following a standardized technique (GE Healthcare Signa HDxt 3.0 T). Images of high-resolution T1-weighted 3D MPRAGE scans of the entire brain (TR = 2500 ms, TE = 2.63 ms, flip angle = 7°, FOV = 256 mm, isotropic voxels 1 × 1 × 1 mm) were acquired as anatomical references. T2 Fluid attenuated inversion recovery (FLAIR) was obtained with the following parameters: TR, 9102 ms; TE, 168.7 ms; matrix, 288,224; section-thickness, 5 mm; FOV, 240 mm. The echo planar imaging diffusion-weighted sequences were collected in the axial plane (TR = 3927 ms, TE = 106 ms, slice thickness = 5 mm; one with *b* = 0 and three orthogonal gradient directions at *b* = 1000).

**Figure 1.**
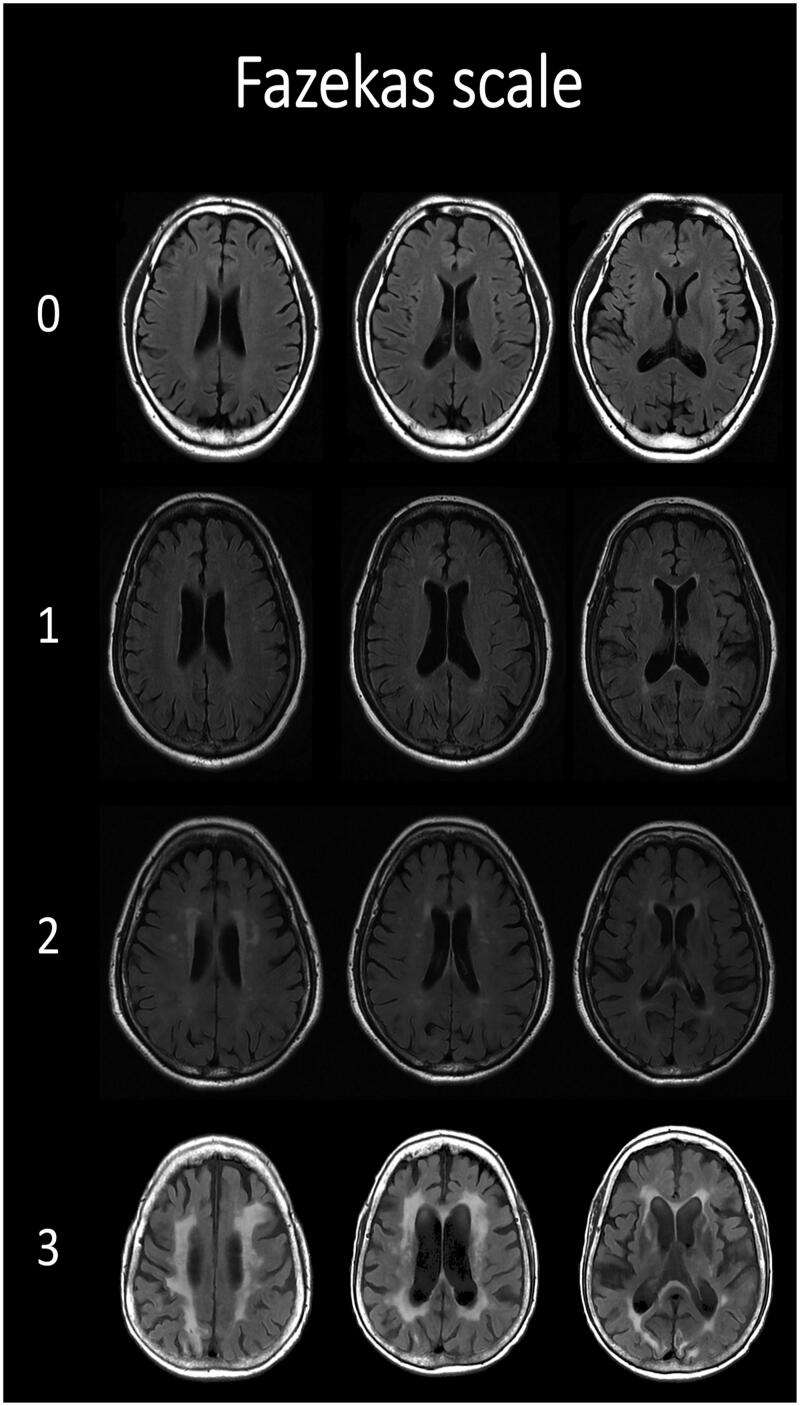
Fazekas scale of periventricular white matter hyperintensities in MRI examples.

WMHs were quantified according to the Fazekas scale [10], and MRI scans were randomly assigned to the two experienced neurologists who were blind to the specific diagnosis to rate the scale. The weighted kappa using inter-rater reliability was 0.84 (0.74, 0.94), 0.78 (0.67, 0.89) in PVWMHs and DWMHs respectively. The Fazekas scale ranges from zero to three, and the value reflects the overall burden of cerebral WMHs. PVWMHs and DWMHs were evaluated separately. PVWMHs scoring 0, 1, 2, and 3 represented absence, caps or pencil-thin lining, smooth halo, and irregular lesions extending into the deep white matter, respectively (Figure 1). DWMHs scoring 0, 1, 2, and 3 represented absence, punctate foci, beginning confluence of foci, and large confluent areas, respectively.

### Cognitive function assessment

Neuropsychological tests were performed by trained psychologists during the HD session. The MMSE containing items of orientation, registration, attention, calculation, recall, language, and praxis is the most widely used screening tool for cognitive function impairment. The maximum score is 30 and cognitive function impairment is defined as a score <24 [18–20].

The MoCA, which is more sensitive to the detection of cognitive impairments in chronic patients undergoing HD, includes the items cube copying, clock drawing, naming, digit-span backward and forward, serial subtraction, selective attention, sentence repetition, phonemic word fluency, verbal abstraction, a 5-word learning and delayed recall task, and spatial and temporal orientation. The tasks comprise multiple domains of cognition, including short-term memory, visuospatial abilities, executive functions, language, attention, concentration and working memory, and orientation to time and place. The maximum score is 30 and cognitive impairment is defined as a score ≤24, the optimal cut-off score for detecting cognitive impairment [21].

The CASI, designed for assessing a broad range of cognitive domains, is a 40-item global cognitive test containing nine cognitive domains including long-term memory, short-term memory, orientation, attention, mental manipulation, list-generating fluency, language, abstraction/judgment, and drawing. The definition of cognitive function impairment is divided into three categories based on age and educational status [22]. The cut-off values for no formal education received, 1–5 years of schooling, and 6 or more years of education were CASI < 50, CASI < 68, and CASI < 80, respectively.

### Statistical analysis

All statistical analyses were performed using SAS version 9.4. Data were described as mean, standard deviation (SD), percentages, median, and quartiles. One-way ANOVA, student’s *t*-tests, or Mann-Whitney U-tests were applied to compare the means of continuous variables and the Chi-square test was used to examine categorical data. The agreement between subjective ratings by two raters was evaluated by inter-rater reliability analysis. Ordinal regression analysis or logistic regression model was used to identify the association between the cognitive function tests and Fazekas scale of PVWMHs and DWMHs after adjusting for confounders (age, sex, comorbidity of diabetes mellitus and cerebrovascular disease, and clinical laboratory data of hemoglobin, albumin, and Kt/V). The results were presented as odds ratios (OR) with 95% confidence intervals (CIs) and a two-tailed *p* < .05 was considered statistically significant.

## Results

### Demographic and clinical characteristics

A total of 59 patients undergoing HD were enrolled in this study and their baseline and clinical characteristics stratified by the Fazekas scale of PVWMHs and DWMHs were presented in Table 1. PVWMHs and DWMHs were evaluated by the Fazekas scale separately (Table 1) and there were no significant differences in gender, comorbidities, blood examinations, or single pool Kt/V between PVWMH and DWMHs groups. The mean age was significantly lower in grade 0 than in other groups in the PVWMHs group (*p* = .001). For the sensitivity analysis, the Fazekas scale was categorized into two groups. The baseline characteristics categorized into two groups by PVWMHs and DWMHs were presented in Supplementary Table 1. The mean age (±SD) was significantly lower in grade 0/1 than in grade 2/3 defined by PVWMHs (58.4 ± 12.9 and 65.5 ± 8.4 respectively, *p* = .019).

**Table 1.** Baseline characteristics of hemodialysis participants stratified by Fazekas scale of periventricular white matter hyperintensities and deep white matter hyperintensities.

	Periventricular hyperintensities (Total *N* = 59)					Deep white matter hyperintensities (Total *N* = 59)				
	Grade 0 *N* = 4	Grade 1 *N* = 23	Grade 2 *N* = 23	Grade 3 *N* = 9	*p*-value	Grade 0 *N* = 8	Grade 1 *N* = 19	Grade 2 *N* = 24	Grade 3 *N* = 8	*p*-value
Age (years)	41.8 ± 4.3	61.3 ± 11.6	65.1 ± 9.0	66.6 ± 6.9	**<.001**	56.0 ± 16.5	61.1 ± 10.9	64.6 ± 7.1	64.3 ± 14.6	.260
Male	2 (50.0%)	10 (43.5%)	14 (60.9%)	4 (44.4%)	.647	4 (50.0%)	8 (42.1%)	15 (62.5%)	3 (37.5%)	.522
Diabetes mellitus	3 (75.0%)	12 (52.2%)	17 (73.9%)	6 (66.7%)	.487	4 (50.0%)	10 (52.6%)	19 (79.2%)	5 (62.5%)	.210
Cerebrovascular disease	0 (0.0%)	5 (21.7%)	8 (34.8%)	2 (22.2%)	.596	1 (12.5%)	4 (21.1%)	7 (29.2%)	3 (37.5%)	.687
Hemoglobin (mg/dl)	11.2 ± 0.1	10.9 ± 1.2	10.7 ± 1.4	10.3 ± 0.9	.552	10.9 ± 0.5	10.5 ± 1.0	11.0 ± 1.5	10.4 ± 0.9	.558
Albumin (mg/dl)	3.9 ± 0.4	3.8 ± 0.2	3.9 ± 0.3	3.7 ± 0.3	.394	3.8 ± 0.4	3.8 ± 0.2	3.9 ± 0.2	3.8 ± 0.3	.884
Single pool Kt/V	1.4 ± 0.3	1.5 ± 0.2	1.5 ± 0.2	1.5 ± 0.2	.746	1.5 ± 0.2	1.6 ± 0.2	1.5 ± 0.2	1.5 ± 0.2	.289

### Cognitive performance across the Fazekas scale

**Figure 2.**
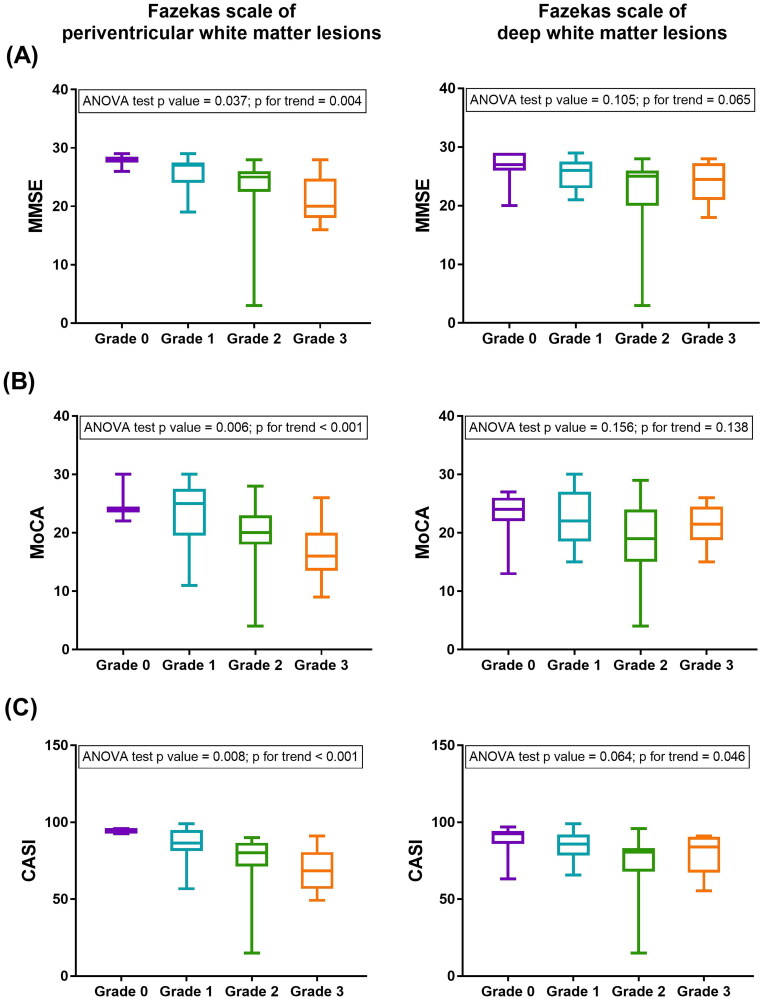
The mean cognitive function test scores across the Fazekas scale of periventricular white matter hyperintensities and deep white matter hyperintensities. (A) Mini-mental State Examination (MMSE), (B) Montreal cognitive assessment (MoCA), and (C) The cognitive Abilities Screening Instrument (CASI).

The mean values of the MMSE, MoCA and CASI scores were compared across the Fazekas scale defined by PVWMHs and DWMHs (Figure 2). There were significant differences in three cognitive function tests across the Fazekas scale of PVWMHs (*p* = .037, .006, and .008 for MMSE, MoCA, and CASI, respectively). The higher scale of PVWMHs was associated with a lower cognitive function (*p* for trend = .004, <.001, and <.001 for MMSE, MoCA, and CASI, respectively). In the sensitivity analysis with two groups of Fazekas scales, three cognitive function tests persistently showed significant differences across the PVWMHs (*p* = .004, .001, <.001 for MMSE, MoCA, and CASI, respectively, Supplemental Figure 1) and of DWMHs (*p* = .013, .036, and .008 for MMSE, MoCA, and CASI, respectively, Supplementary Figure 1). In summary, a higher Fazekas scale score, particularly defined in PVWMHs, strongly correlated with lower cognitive test results.

### Subdomain of the CASI and Fazekas scale

**Figure 3.**
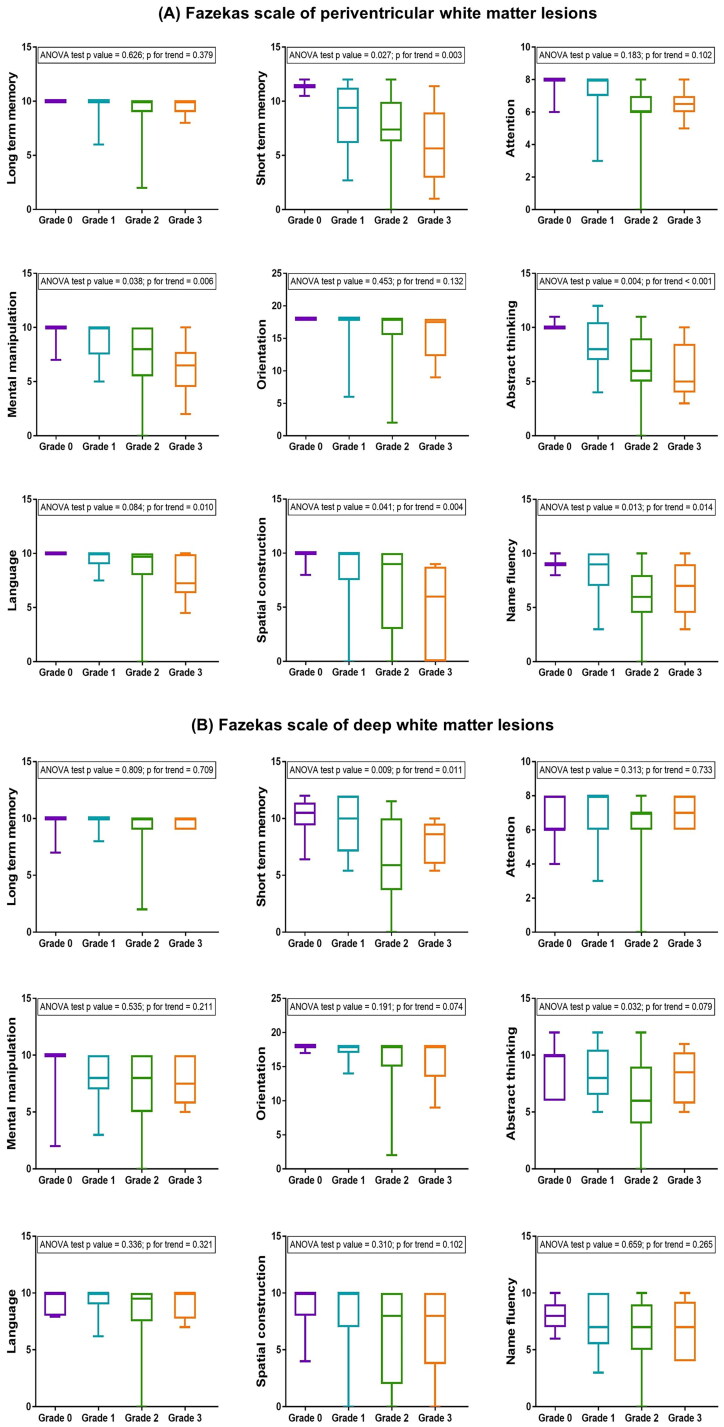
The mean values of the cognitive Abilities Screening Instrument (CASI) subdomains across the Fazekas scale of (A) periventricular white matter hyperintensities and (B) deep white matter hyperintensities.

The mean values of CASI subdomains were compared across Fazekas scales defined by PVWMHs and DWMHs (Figure 3). Higher grades were linked to poor performance in subdomains of short-term memory, mental manipulation, abstract thinking, language, spatial construction, and name fluency (*p* = .003, .006, <.001, .010, .004, .014, respectively, Figure 3). Among the grades of DWMHs, fewer subdomains revealed obvious correspondence, with only abstract thinking and short-term memory showing a significant difference (*p* = .032 and .009, respectively), and short-term memory was associated with higher grades on the Fazekas scale (*p* for trend = .011). Overall, deficits in short-term memory, mental manipulation, abstract thinking, language, spatial construction, and name fluency in the CASI correlated with higher Fazekas scale scores and showed a trend defined by PVWMHs. However, only short-term memory demonstrated a trend associated with the Fazekas scale of DWMHs.

In the sensitivity analysis with two groups of the Fazekas scale, most CASI subdomains including short-term memory, attention, mental manipulation, abstract thinking, language, spatial construction, and name fluency, showed significant differences in comparisons across the Fazekas scale of PVWMHs (*p* = .020, .034, .003, <.001, .013, .009, .002, respectively, Supplementary Figure 2), whereas only short-term memory, orientation, and abstract thinking revealed significant differences across the Fazekas scale of DWMHs (*p* = .002, .022, .012, respectively, Supplementary Figure 2). To summarize, a higher Fazekas scale of PVWMHs displayed strong correlations with poor cognitive performances in the CASI subdomains.

### Cognitive functions were associated with the Fazekas scale

The ordinal regression analysis or logistic regression model showed that three cognitive function tests were persistently associated with the Fazekas score of PVWMHs after adjusting for confounders in models 1–3 (Table 2). However, neither cognitive function test presented an association with or without adjustment in the DWMHs. In summary, the higher the Fazekas score of PVWMHs, the poorer the patient’s cognitive performance.

**Table 2.** Association between cognitive function score and Fazekas scale of periventricular white matter hyperintensities (PVWMHs) and deep white matter hyperintensities (DWMHs) in hemodialysis participants using ordinal regression analysis with confounders adjustment.

	Odds ratio (95% CI)		
	Model 1	Model 2	Model 3
PVWMHs			
MMSE	0.86 (0.76, 0.97)*	0.87 (0.77, 0.98)*	0.87 (0.76, 0.99)*
MoCA	0.84 (0.75, 0.93)*	0.86 (0.77, 0.95)*	0.86 (0.76, 0.97)*
CASI	0.95 (0.91, 0.98)*	0.95 (0.92, 0.98)*	0.95 (0.92, 0.99)*
Long term memory	0.86 (0.59, 1.25)	0.88 (0.60, 1.30)	0.94 (0.63, 1.40)
Short term memory	0.77 (0.65, 0.92)*	0.78 (0.64, 0.94)*	0.81 (0.66, 1.00)
Attention	0.77 (0.55, 1.06)	0.81 (0.57, 1.14)	0.87 (0.61, 1.25)
Mental manipulation	0.77 (0.63, 0.94)*	0.80 (0.65, 0.98)*	0.79 (0.63, 0.99)*
Orientation	0.90 (0.78, 1.04)	0.92 (0.79, 1.06)	0.91 (0.78, 1.07)
Abstract thinking	0.69 (0.56, 0.85)*	0.71 (0.57, 0.90)*	0.70 (0.54, 0.92)*
Language	0.72 (0.54, 0.96)*	0.74 (0.55, 1.00)	0.76 (0.55, 1.06)
Spatial construction	0.81 (0.69, 0.94)*	0.79 (0.67, 0.93)*	0.78 (0.64, 0.94)*
Name fluency	0.75 (0.59, 0.94)*	0.80 (0.63, 1.01)	0.77 (0.59, 1.01)
DWMHs			
MMSE	0.91 (0.82, 1.01)	0.92 (0.82, 1.02)	0.89 (0.79, 1.01)
MoCA	0.93 (0.85, 1.02)	0.95 (0.86, 1.04)	0.91 (0.81, 1.03)
CASI	0.97 (0.94, 1.00)	0.97 (0.94, 1.00)	0.96 (0.93, 1.00)
Long term memory	0.92 (0.63, 1.34)	0.95 (0.65, 1.40)	1.00 (0.67, 1.48)
Short term memory	0.81 (0.69, 0.96)*	0.82 (0.68, 0.98)*	0.77 (0.63, 0.95)*
Attention	0.92 (0.66, 1.26)	0.97 (0.69, 1.35)	1.01 (0.71, 1.44)
Mental manipulation	0.88 (0.73, 1.06)	0.90 (0.74, 1.09)	0.92 (0.74, 1.14)
Orientation	0.88 (0.76, 1.02)	0.89 (0.77, 1.03)	0.88 (0.75, 1.04)
Abstract thinking	0.83 (0.69, 1.01)	0.86 (0.70, 1.06)	0.85 (0.67, 1.09)
Language	0.87 (0.67, 1.13)	0.89 (0.68, 1.18)	0.86 (0.62, 1.18)
Spatial construction	0.88 (0.76, 1.02)	0.88 (0.75, 1.02)	0.83 (0.69, 1.00)
Name fluency	0.89 (0.72, 1.10)	0.93 (0.74, 1.17)	0.86 (0.67, 1.11)

Model 1: crude model.

Model 2: adjusted for age, sex.

Model 3: adjusted for age, sex, comorbidity (diabetes mellitus and cerebrovascular disease), and clinical laboratory data (hemoglobin, albumin, and Kt/V).

*p < .05.

Among the CASI subdomains in the PVWMHs, short-term memory, mental manipulation, abstract thinking, language, spatial construction, and name fluency were associated with the Fazekas scale in the crude model (model 1, Table 2). However, the associations attenuated after adjusting for confounding factors in model 3, with significant associations in the mental manipulation, abstract thinking, and spatial construction subdomains (odds ratio (95% CI) = 0.79 (0.63, 0.99), 0.71 (0.54, 0.92), 0.78 (0.64, 0.95), respectively). Nevertheless, in the DWMHs evaluation, only short-term memory revealed a significant association with the Fazekas scale in model 3 (odds ratio (95% CI) = 0.78 (0.63, 0.96)). Consistent with the previous results, the associations between cognitive function performance and the Fazekas scale were more predominant in the PVWMHs.

## Discussion

In this cross-sectional study, increased Fazekas scale scores, particularly within PVWMHs, demonstrated an association with diminished cognitive function as indicated by the MMSE, MoCA, and CASI assessments. Additionally, the grades displayed noticeable links with poor performance in CASI subdomains.

The overall cognition profiles provided by this research suggested that higher grades of the Fazekas scale in PVWMHs were strongly associated with lower cognitive function, which was partially in line with previous findings in patients with Parkinson’s disease [17,23]. However, the comprehensive confounding factors in the statistical model have not been adequately accounted for. In the case of the effect of cerebrovascular disease [13,16], we adjusted for the comorbidities of cerebrovascular disease but the correlation remained significant. Moreover, deficits in various CASI subdomains such as short-term memory, attention, mental manipulation, abstract thinking, language, spatial construction, and name fluency, correlated with higher Fazekas scale scores, especially in PVWMHs. The subdomains including memory, language, and name fluency were similar in previous research focusing on the CKD and ESKD population [5–8]. Multiple factors including cerebrovascular diseases, albuminuria, anaemia, secondary hyperparathyroidism, electrolyte imbalance, uremic toxins, inflammation and malnutrition contributed to the cognitive dysfunction [24–26]. Though different cognitive assessment tools were used, cognitive impairment was evident in patients with WMHs [11,15,17,23,27]. On the other hand, despite the relationship between Alzheimer’s disease and the Fazekas scale [27,28], the recent ARIC study [29] suggested that glomerular filtration rate (GFR) and urinary albumin-creatinine ratio (UACR) were associated with structural brain damage but not the region affected by Alzheimer’s disease. Thus, a higher Fazekas scale in patients undergoing HD indicating more structural brain damage might suggest a more serious cognitive decline.

White matter hyperintensities (WMHs), also referred to as leukoaraiosis, are common neuroradiological abnormalities in the elderly [30]. Linked to cerebral small vessel disease, WMHs have been associated with an increased risk of cognitive dysfunction and all-cause dementia [31], probably by disrupting the connections between the cortex and the subcortical nuclei [32,33] or contributing to the accumulation of amyloid-β proteins [34]. On the other hand, the relationship between GFR and concluded that CKD is associated with poorer WMHs [25,35,36], with mechanisms related to cerebrovascular disease, systemic inflammation, cerebral hypoperfusion, oxidative stress, disturbances of small blood vessels, breakdown of the blood-brain barrier, glial activation, loss of oligodendrocytes, and demyelination caused by chronic diffuse hypoperfusion [37–39].

Interestingly, cognitive impairment was predominant in PVWMHs rather than DWMHs. Claus et al. [14] claimed that WMHs had a low impact on cognitive functions and suggested that certain cognitive impairments, particularly memory, stem from medial temporal atrophy (MTA) since MTA is related to both WMHs and memory function [40] and the relationship between WMHs and memory function was also not observed in several previous studies [41–43]. Another study also supported the association between vascular dementia and increased DWMHs [44] but other research [45,46] revealed that WMH volume measurement could be more sensitive than traditional visual scales such as the Fazekas scale and Scheltens Scale [47]. The LADIS study [48] stated that the ceiling effect of the visual scales and the poor discrimination of absolute lesion volumes contributed to the poor sensitivity. The method to define PVWMHs and DWMHs is in dispute and different classification schemes have been suggested [49]. Griffitani et al. [50] suggested that the classification criteria of PVWMHs and DWMHs were not a major obstacle to study comparison. The white matter measures used in the current research may not be the most optimal; however, several studies indicated similar outcomes as PV burden related to the development of cognitive impairment [51–53].

The causes of the differing effects of PVWMHs and DWMHs on cognitive function remain unclear. DWMHs primarily interfere with short-looped U-fibers, which connect neighbouring cortical areas. On the other hand, PVWMHs impact the long association fibres that connect the cortex to subcortical nuclei [54]. Subcortical brain structures are widely considered pivotal for the speed of cognitive processes and memory function [55]. Consequently, lesions in PVWMHs could disrupt critical neural networks, leading to cognitive decline. Moreover, PVWMHs have the potential to disturb PV cholinergic fibres that link the nucleus basalis to the cerebral cortex [56], thereby contributing to cognitive impairment. Altogether, PVWMHs serve as predictors of cognitive decline within the scope of this study. Accordingly, these results explain why PVWMHs can predict cognition decline.

The present study has high clinical value due to the limited studies on cognitive function in CKD patients. As CKD becomes a more pressing public concern, studies have evaluated the relationship between GFR and UACR with structural brain damage [29]. A recent study [57] also investigated the relationship between structural changes in the brain using functional MRI images and cognitive impairment in patients undergoing HD. However, to our knowledge, there has been no research connecting structural brain changes measured with the Fazekas scale and cognitive performance specifically in patients undergoing HD. Moreover, we included the commonly used MMSE and MoCA tests as well as the CASI for detailed subdomain analysis.

There are some limitations in the present study. First, the sample size was relatively small, although statistical analysis showed significance. Second, this study was a cross-sectional design, so it was difficult to clarify the definite order of cognition decline, HD, and the structural changes in the brain because they were simultaneously assessed. Third, the lack of a matched control group for comparison was another limitation. Moreover, since it was difficult to accurately assess the brain lesions, the rating technique chosen may affect the outcome. The present study reported an inter-rater reliability of over 0.75 evaluated by two neurologists for the Fazekas scale despite its reader-dependent nature. Finally, other potential confounders such as other comorbidities that may affect the result were not taken into consideration.

## Conclusion

In conclusion, the present study revealed an inverse correlation between the Fazekas scale and cognition in patients undergoing HD. The Fazekas scale of PVWMHs was associated with various CASI subdomains such as memory, language, and name fluency. The association between the Fazekas scale and PVWMHs was found to be stronger compared to that of DWMHs. Further investigations are required to validate these results and to clarify the underlying mechanisms.

## Supplementary Material

Supplemental Material

## Data Availability

The data will be provided upon request to the corresponding author.

## References

[1] Stevens LA, Viswanathan G, Weiner DE. Chronic kidney disease and end-stage renal disease in the elderly ­population: current prevalence, future projections, and clinical significance. Adv Chronic Kidney Dis. 2010;17(4):1–12. doi: 10.1053/j.ackd.2010.03.010. 20610356 PMC3160131

[2] Hannan M, Steffen A, Quinn L, et al. The assessment of cognitive function in older adult patients with chronic kidney disease: an integrative review. J Nephrol. 2019;32(2):211–230. doi: 10.1007/s40620-018-0494-2. 29802584 PMC8174670

[3] Vanderlinden JA, Ross-White A, Holden R, et al. Quantifying cognitive dysfunction across the spectrum of end-stage kidney disease: a systematic review and meta-analysis. Nephrology. 2019;24(1):5–16. doi: 10.1111/nep.13448. 30094890

[4] Drew DA, Tighiouart H, Rollins J, et al. Evaluation of screening tests for cognitive impairment in patients receiving maintenance hemodialysis. J Am Soc Nephrol. 2020;31(4):855–864. doi: 10.1681/ASN.2019100988. 32132197 PMC7191919

[5] Yaffe K, Ackerson L, Kurella Tamura M, et al. Chronic kidney disease and cognitive function in older adults: findings from the chronic renal insufficiency cohort cognitive study. J Am Geriatr Soc. 2010;58(2):338–345. doi: 10.1111/j.1532-5415.2009.02670.x. 20374407 PMC2852884

[6] Berger I, Wu S, Masson P, et al. Cognition in chronic kidney disease: a systematic review and meta-analysis. BMC Med. 2016;14(1):206. doi: 10.1186/s12916-016-0745-9. 27964726 PMC5155375

[7] Murray AM, Bell EJ, Tupper DE, et al. The brain in kidney disease (BRINK) cohort study: design and baseline cognitive function. Am J Kidney Dis. 2016;67(4):593–600. doi: 10.1053/j.ajkd.2015.11.008. 26744128 PMC5271565

[8] O'Lone E, Connors M, Masson P, et al. Cognition in people with End-Stage kidney disease treated with hemodialysis: a systematic review and meta-analysis. Am J Kidney Dis. 2016;67(6):925–935. doi: 10.1053/j.ajkd.2015.12.028. 26919914

[9] Erten-Lyons D, Woltjer R, Kaye J, et al. Neuropathologic basis of white matter hyperintensity accumulation with advanced age. Neurology. 2013;81(11):977–983. doi: 10.1212/WNL.0b013e3182a43e45. 23935177 PMC3888199

[10] Fazekas F, Chawluk JB, Alavi A, et al. MR signal abnormalities at 1.5 T in alzheimer’s dementia and normal aging. AJR Am J Roentgenol. 1987;149(2):351–356. doi: 10.2214/ajr.149.2.351. 3496763

[11] Sudo FK, Alves CE, Alves GS, et al. White matter hyperintensities, executive function and global cognitive performance in vascular mild cognitive impairment. Arq Neuropsiquiatr. 2013;71(7):431–436. doi: 10.1590/0004-282X20130057. 23857627

[12] Wieczorek J, Mizia-Stec K, Lasek-Bal A, et al. CHA2DS2-Vasc score, age and body mass index as the main risk factors of hyperintense brain lesions in ­asymptomatic patients with paroxysmal non-valvular atrial fibrillation. Int J Cardiol. 2016;215:476–481. doi: 10.1016/j.ijcard.2016.04.094. 27131768

[13] Li J, Zhao Y, Mao J. Association between the extent of white matter damage and early cognitive impairment following acute ischemic stroke. Exp Ther Med. 2017;13(3):909–912. doi: 10.3892/etm.2017.4035. 28450918 PMC5403345

[14] Claus JJ, Coenen M, Staekenborg SS, et al. Cerebral white matter lesions have low impact on cognitive function in a large elderly memory clinic population. J Alzheimers Dis. 2018;63(3):1129–1139. doi: 10.3233/JAD-171111. 29710708

[15] Kynast J, Lampe L, Luck T, et al. White matter hyperintensities associated with small vessel disease impair ­social cognition beside attention and memory. J Cereb Blood Flow Metab. 2018;38(6):996–1009. doi: 10.1177/0271678X17719380. 28685621 PMC5999004

[16] Fruhwirth V, Enzinger C, Fandler-Höfler S, et al. Baseline white matter hyperintensities affect the course of cognitive function after small vessel disease-related stroke: a prospective observational study. Eur J Neurol. 2021;28(2):401–410. doi: 10.1111/ene.14593. 33065757 PMC7839458

[17] Agarwal P, Panda AK, Jena S, et al. Correlation of cerebral atrophy and white matter hyperintensity burden in MRI with clinical cognitive decline. Siriraj Med J. 2022;74(5):323–329. doi: 10.33192/Smj.2022.39.

[18] Odagiri G, Sugawara N, Kikuchi A, et al. Cognitive function among hemodialysis patients in Japan. Ann Gen Psychiatry. 2011;10(1):20. doi: 10.1186/1744-859X-10-20. 21867512 PMC3171713

[19] Jung S, Lee YK, Choi SR, et al. Relationship between cognitive impairment and depression in dialysis patients. Yonsei Med J. 2013;54(6):1447–1453. doi: 10.3349/ymj.2013.54.6.1447. 24142650 PMC3809877

[20] Fadili W, Al Adlouni A, Louhab N, et al. Prevalence and risk factors of cognitive dysfunction in chronic hemodialysis patients. Aging Ment Health. 2014;18(2):207–211. doi: 10.1080/13607863.2013.823375. 23906058

[21] Tiffin-Richards FE, Costa AS, Holschbach B, et al. The montreal cognitive assessment (MoCA) - a sensitive screening instrument for detecting cognitive impairment in chronic hemodialysis patients. PLOS One. 2014;9(10):e106700. doi: 10.1371/journal.pone.0106700. 25347578 PMC4209968

[22] Lin KN, Wang PN, Liu CY, et al. Cutoff scores of the cognitive abilities screening instrument, chinese version in screening of dementia. Dement Geriatr Cogn Disord. 2002;14(4):176–182. doi: 10.1159/000066024. 12411759

[23] Vasconcellos LF, Pereira JS, Adachi M, et al. Correlation of MRI visual scales with neuropsychological profile in mild cognitive impairment of parkinson’s disease. Parkinsons Dis. 2017;2017:7380102. doi: 10.1155/2017/7380102. 28409050 PMC5376452

[24] Miyazaki S, Kitamura M, Hayashida M, et al. Survival and cognitive deterioration in elderly patients undergoing hemodialysis. Geriatr Gerontol Int. 2023;23(2):111–116. doi: 10.1111/ggi.14531. 36608644

[25] Wei CS, Yan CY, Yu XR, et al. Association between white matter hyperintensities and chronic kidney disease: a systematic review and Meta-Analysis. Front Med (Lausanne). 2022;9:770184. doi: 10.3389/fmed.2022.770184. 35592851 PMC9112853

[26] Lin YT, Wu PH, Lee HH, et al. Indole-3 acetic acid ­increased risk of impaired cognitive function in patients receiving hemodialysis. Neurotoxicology. 2019;73:85–91. doi: 10.1016/j.neuro.2019.02.019. 30826344

[27] Makino T, Umegaki H, Suzuki Y, et al. Relationship ­between small cerebral white matter lesions and cognitive function in patients with alzheimer’s disease and amnestic mild cognitive impairment. Geriatr Gerontol Int. 2014;14(4):819–826. doi: 10.1111/ggi.12176. 24215176

[28] Mimenza-Alvarado A, Aguilar-Navarro SG, Yeverino-Castro S, et al. Neuroimaging characteristics of small-vessel disease in older adults with normal cognition, mild cognitive impairment, and alzheimer disease. Dement Geriatr Cogn Dis Extra. 2018;8(2):199–206. doi: 10.1159/000488705. 29928288 PMC6006607

[29] Scheppach JB, Wu A, Gottesman RF, et al. Association of kidney function measures with signs of neurodegeneration and small vessel disease on brain magnetic resonance imaging: the atherosclerosis risk in communities (ARIC) study. Am J Kidney Dis. 2023;81(3):261–269.e1. doi: 10.1053/j.ajkd.2022.07.013. 36179945 PMC9974563

[30] Garnier-Crussard A, Desestret V, Cotton F, et al. White matter hyperintensities in ageing: pathophysiology, associated cognitive disorders and prevention. Rev Med Interne. 2020;41(7):475–484. doi: 10.1016/j.revmed.2020.02.009. 32122680

[31] Thorn LM, Shams S, Gordin D, et al. Clinical and MRI features of cerebral small-vessel disease in type 1 diabetes. Diabetes Care. 2019;42(2):327–330. doi: 10.2337/dc18-1302. 30552131

[32] Debette S, Schilling S, Duperron MG, et al. Clinical significance of magnetic resonance imaging markers of vascular brain injury: a systematic review and meta-analysis. JAMA Neurol. 2019;76(1):81–94. doi: 10.1001/jamaneurol.2018.3122. 30422209 PMC6439887

[33] Thal DR, Attems J, Ewers M. Spreading of amyloid, tau, and microvascular pathology in alzheimer’s disease: findings from neuropathological and neuroimaging studies. J Alzheimers Dis. 2014;42(Suppl 4): s 421–9. doi: 10.3233/JAD-141461. 25227313

[34] van Westen D, Lindqvist D, Blennow K, et al. Cerebral white matter lesions - associations with Aβ isoforms and amyloid PET. Sci Rep. 2016;6(1):20709. doi: 10.1038/srep20709. 26856756 PMC4746584

[35] Vogels SC, Emmelot-Vonk MH, Verhaar HJ, et al. The association of chronic kidney disease with brain lesions on MRI or CT: a systematic review. Maturitas. 2012;71(4):331–336. doi: 10.1016/j.maturitas.2012.01.008. 22318093

[36] Shima H, Ishimura E, Naganuma T, et al. Decreased kidney function is a significant factor associated with silent cerebral infarction and periventricular hyperintensities. Kidney Blood Press Res. 2011;34(6):430–438. doi: 10.1159/000328722. 21709424

[37] Li Q, Yang Y, Reis C, et al. Cerebral small vessel disease. Cell Transplant. 2018;27(12):1711–1722. doi: 10.1177/0963689718795148. 30251566 PMC6300773

[38] De Silva TM, Miller AA. Cerebral small vessel disease: targeting oxidative stress as a novel therapeutic strategy? Front Pharmacol. 2016;7:61. doi: 10.3389/fphar.2016.00061. 27014073 PMC4794483

[39] Pantoni L. Cerebral small vessel disease: from pathogenesis and clinical characteristics to therapeutic challenges. Lancet Neurol. 2010;9(7):689–701. doi: 10.1016/S1474-4422(10)70104-6. 20610345

[40] de Leeuw FE, Barkhof F, Scheltens P. White matter ­lesions and hippocampal atrophy in Alzheimer’s disease. Neurology. 2004;62(2):310–312. doi: 10.1212/01.wnl.0000103289.03648.ad. 14745078

[41] Muller M, Appelman APA, van der Graaf Y, et al. Brain atrophy and cognition: interaction with cerebrovascular pathology? Neurobiol Aging. 2011;32(5):885–893. doi: 10.1016/j.neurobiolaging.2009.05.005. 19520460

[42] Carmichael O, Mungas D, Beckett L, et al. MRI predictors of cognitive change in a diverse and carefully characterized elderly population. Neurobiol Aging. 2012;33(1):83–95. doi: 10.1016/j.neurobiolaging.2010.01.021. 20359776 PMC2909327

[43] Birdsill AC, Koscik RL, Jonaitis EM, et al. Regional white matter hyperintensities: aging, Alzheimer’s disease risk, and cognitive function. Neurobiol Aging. 2014;35(4):769–776. doi: 10.1016/j.neurobiolaging.2013.10.072. 24199958 PMC3880609

[44] Smith CD, Johnson ES, Van Eldik LJ, et al. Peripheral (deep) but not periventricular MRI white matter hyperintensities are increased in clinical vascular dementia compared to Alzheimer’s disease. Brain Behav. 2016;6(3):e00438. 26925303 10.1002/brb3.438 PMC4754499

[45] Prins ND, van Straaten EC, van Dijk EJ, et al. Measuring progression of cerebral white matter lesions on MRI: visual rating and volumetrics. Neurology. 2004;62(9):1533–1539. doi: 10.1212/01.wnl.0000123264.40498.b6. 15136677

[46] van Straaten EC, Fazekas F, Rostrup E, et al. Impact of white matter hyperintensities scoring method on correlations with clinical data: the LADIS study. Stroke. 2006;37(3):836–840. doi: 10.1161/01.STR.0000202585.26325.74. 16439704

[47] Scheltens P, Leys D, Barkhof F, et al. Atrophy of medial temporal lobes on MRI in “probable” Alzheimer’s disease and normal ageing: diagnostic value and neuropsychological correlates. J Neurol Neurosurg Psychiatry. 1992;55(10):967–972. doi: 10.1136/jnnp.55.10.967. 1431963 PMC1015202

[48] Gouw AA, van der Flier WM, van Straaten EC, et al. Reliability and sensitivity of visual scales versus volumetry for evaluating white matter hyperintensity progression. Cerebrovasc Dis. 2008;25(3):247–253. doi: 10.1159/000113863. 18216467

[49] Kim KW, MacFall JR, Payne ME. Classification of white matter lesions on magnetic resonance imaging in ­elderly persons. Biol Psychiatry. 2008;64(4):273–280. doi: 10.1016/j.biopsych.2008.03.024. 18471801 PMC2593803

[50] Griffanti L, Jenkinson M, Suri S, et al. Classification and characterization of periventricular and deep white matter hyperintensities on MRI: a study in older adults. Neuroimage. 2018;170:174–181. doi: 10.1016/j.neuroimage.2017.03.024. 28315460

[51] De Groot JC, De Leeuw FE, Oudkerk M, et al. Periventricular cerebral white matter lesions predict rate of cognitive decline. Ann Neurol. 2002;52(3):335–341. doi: 10.1002/ana.10294. 12205646

[52] van den Heuvel DM, ten Dam VH, de Craen AJ, et al. Increase in periventricular white matter hyperintensities parallels decline in mental processing speed in a non-demented elderly population. J Neurol Neurosurg Psychiatry. 2006;77(2):149–153. doi: 10.1136/jnnp.2005.070193. 16421114 PMC2077562

[53] Silbert LC, Howieson DB, Dodge H, et al. Cognitive impairment risk: white matter hyperintensity progression matters. Neurology. 2009;73(2):120–125. doi: 10.1212/WNL.0b013e3181ad53fd. 19597134 PMC2713187

[54] Owen JP, Wang MB, Mukherjee P. Periventricular white matter is a nexus for network connectivity in the human brain. Brain Connect. 2016;6(7):548–557. doi: 10.1089/brain.2016.0431. 27345586

[55] Darvesh S, Freedman M. Subcortical dementia: a neurobehavioral approach. Brain Cogn. 1996;31(2):230–249. doi: 10.1006/brcg.1996.0043. 8812000

[56] Selden NR, Gitelman DR, Salamon-Murayama N, et al. Trajectories of cholinergic pathways within the cerebral hemispheres of the human brain. Brain. 1998;121(12):2249–2257. doi: 10.1093/brain/121.12.2249. 9874478

[57] Guo H, Liu W, Li H, et al. Structural and functional brain changes in hemodialysis patients with End-Stage renal disease: DTI analysis results and ALFF analysis results. Int J Nephrol Renovasc Dis. 2021;14:77–86. doi: 10.2147/IJNRD.S295025. 33727853 PMC7955761

