# Supplementary material for: Cerebral white matter burden is linked to cognitive function in patients undergoing hemodialysis
Source: Ann Med. 2024 Feb 7;56(1):2310142. doi: 10.1080/07853890.2024.2310142 (PMC10851831; doi:10.1080/07853890.2024.2310142)

**Supplemental Materials for**

**“Cerebral white matter burden is linked to cognitive function in patients undergoing hemodialysis”**

**Supplementary Table 1.** The baseline characteristics in hemodialysis participants stratified by Grade 0/1 and Grade 2/3 Fazekas scale of periventricular white matter lesions and deep white matter lesions

**Supplementary Figure 1.** The mean cognitive function test scores across the Grade 0/1 and Grade 2/3 Fazekas scale of periventricular white matter lesions and deep white matter lesions. (A) Mini-Mental State Examination (MMSE), (B) Montreal Cognitive Assessment (MoCA), and (C) The Cognitive Abilities Screening Instrument (CASI)

**Supplementary Figure 2.** The mean values of the Cognitive Abilities Screening Instrument (CASI) subdomains across the Grade 0/1 and Grade 2/3 Fazekas scale of periventricular and deep white matter lesions

**Supplementary Table 1.** The baseline characteristics in hemodialysis participants stratified by Grade 0/1 and Grade 2/3 Fazekas scale of periventricular white matter lesions and deep white matter lesions

|  | **Periventricular lesions (Total N = 59)** | | | **Deep white matter lesions  (Total N = 59)** | | |
| --- | --- | --- | --- | --- | --- | --- |
|  | Grade 0 and 1  N = 27 | Grade 2 and 3  N = 32 | *p*-value | Grade 0 and 1  N = 27 | Grade 2 and 3  N = 32 | *p*-value |
| Age (years) | 58.4 ± 12.9 | 65.5 ± 8.4 | **0.019** | 59.6 ± 12.7 | 64.5 ± 9.3 | 0.091 |
| Male | 12 (44.4%) | 18 (56.3%) | 0.521 | 12 (44.4%) | 18 (56.3%) | 0.521 |
| Diabetes mellitus | 15 (55.6%) | 23 (71.9%) | 0.302 | 14 (51.9%) | 24 (75.0%) | 0.115 |
| Cerebrovascular disease | 5 (18.5%) | 10 (31.3%) | 0.413 | 5 (18.5%) | 10 (31.3%) | 0.413 |
| Hemoglobin (mg/dl) | 10.9 ± 1.1 | 10.6 ± 1.2 | 0.233 | 10.7 ± 0.9 | 10.8 ± 1.4 | 0.580 |
| Albumin (mg/dl) | 3.8 ± 0.2 | 3.8 ± 0.3 | 0.735 | 3.8 ± 0.3 | 3.8 ± 0.2 | 0.672 |
| Single pool Kt/V | 1.5 ± 0.2 | 1.5 ± 0.2 | 0.988 | 1.6 ± 0.2 | 1.5 ± 0.2 | 0.110 |

**Supplementary Figure 1.** The mean cognitive function test scores across the Grade 0/1 and Grade 2/3 Fazekas scale of periventricular white matter lesions and deep white matter lesions. (A) Mini-Mental State Examination (MMSE), (B) [Montreal Cognitive Assessment](https://en.wikipedia.org/wiki/Montreal_Cognitive_Assessment) (MoCA), and (C) [The Cognitive Abilities Screening Instrument (CASI)](https://www.ncbi.nlm.nih.gov/pubmed/8054493)


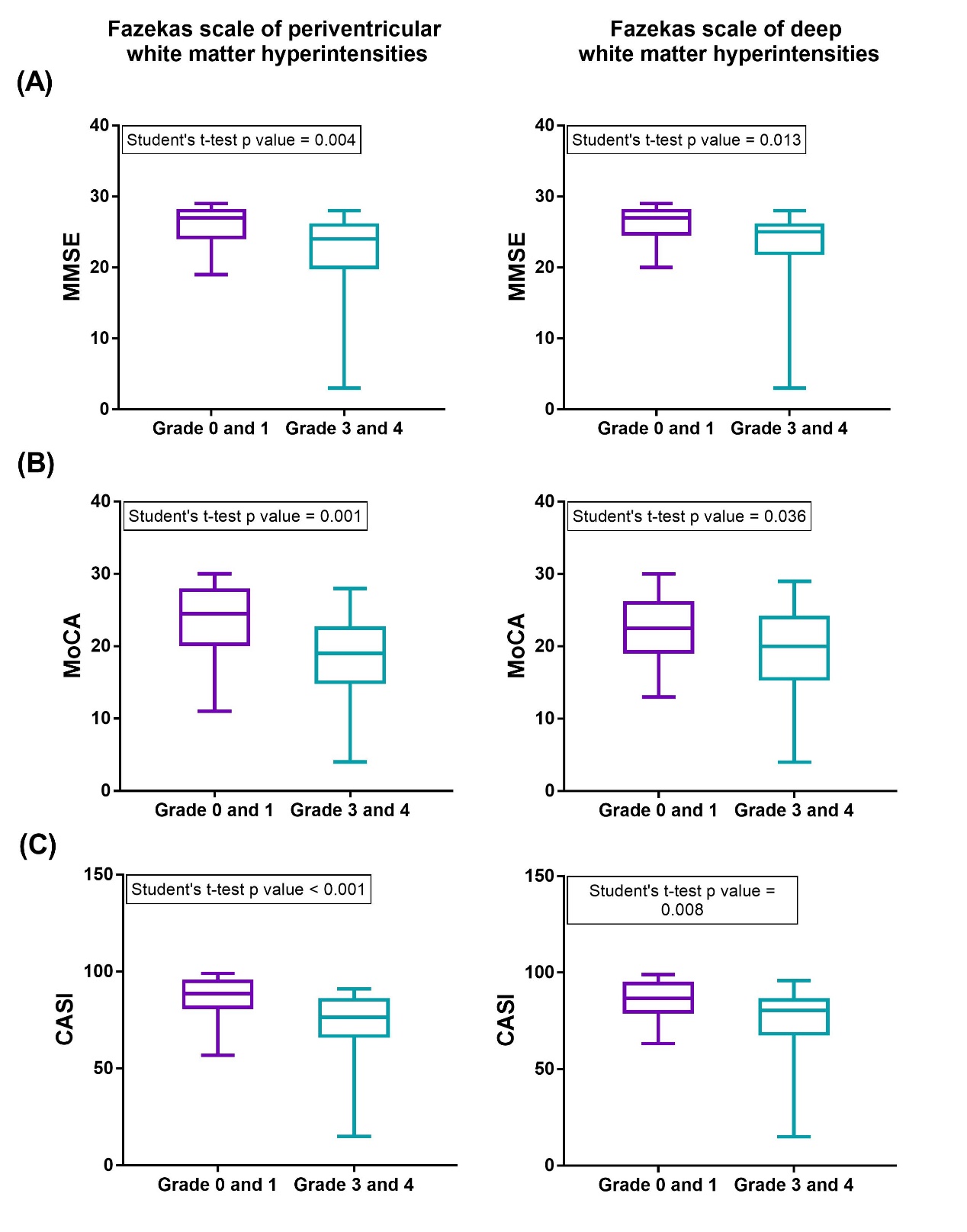


**Supplementary Figure 2.** The mean values of [the Cognitive Abilities Screening Instrument (CASI)](https://www.ncbi.nlm.nih.gov/pubmed/8054493) subdomains across the Grade 0/1 and Grade 2/3 Fazekas scale of periventricular and deep white matter lesions.


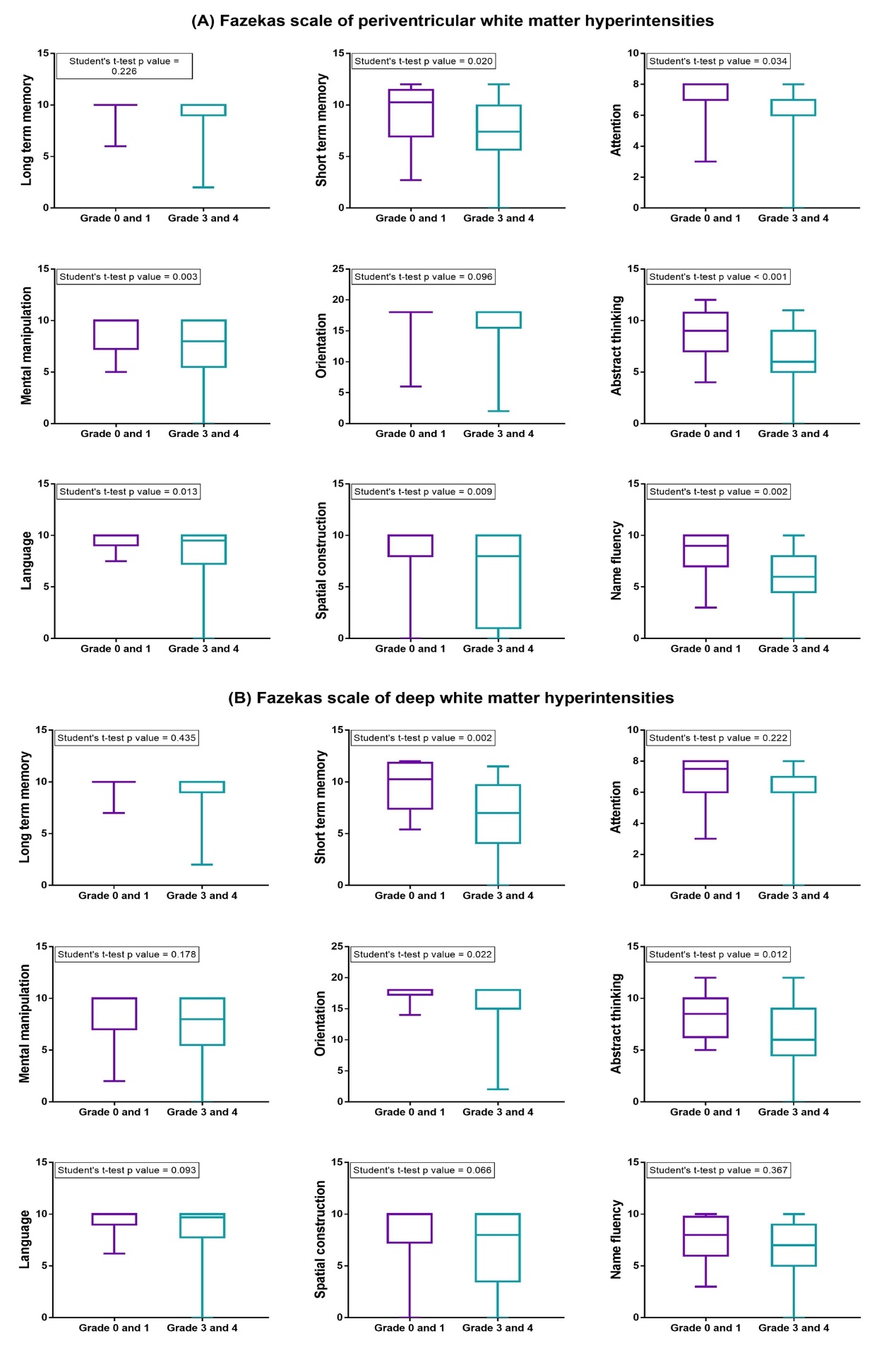

Supplement: Supplemental Material [file IANN_A_2310142_SM3821.docx]
